# Bacteriophage SPO1 protein Gp46 suppresses functions of HU protein in *Francisella tularensis*

**DOI:** 10.3389/fmicb.2023.1330109

**Published:** 2023-12-08

**Authors:** Petra Spidlova, Eliska Sokolova, Pavla Pavlik

**Affiliations:** ^1^Department of Molecular Pathology and Biology, Faculty of Military Health Sciences, University of Defence, Hradec Kralove, Czechia; ^2^Faculty of Pharmacy in Hradec Kralove, Charles University, Hradec Kralove, Czechia

**Keywords:** *Francisella*, HU protein, Gp46, virulence, histone-like protein, transcription factor, nucleoid-associated protein

## Abstract

The nucleoid-associated protein HU is a common bacterial transcription factor, whose role in pathogenesis and virulence has been described in many bacteria. Our recent studies showed that the HU protein is an indispensable virulence factor in the human pathogenic bacterium *Francisella tularensis*, a causative agent of tularemia disease, and that this protein can be a key target in tularemia treatment or vaccine development. Here, we show that *Francisella* HU protein is inhibited by Gp46, a protein of *Bacillus subtilis* bacteriophage SPO1. We predicted that Gp46 could occupy the *F. tularensis* HU protein DNA binding site, and subsequently confirmed the ability of Gp46 to abolish the DNA-binding capacity of HU protein. Next, we showed that the growth of *Francisella* wild-type strain expressing Gp46 *in trans* corresponded to that of a deletion mutant strain lacking the HU protein. Similarly, the efficiency of intracellular proliferation in mouse macrophages resembled that of the deletion mutant strain, but not that of the wild-type strain. These results, in combination with findings from a recent study on Gp46, enabled us to confirm that Gp46 could be a universal inhibitor of HU proteins among bacterial species.

## 1 Introduction

*Francisella tularensis*, a Gram-negative bacterium, is the causative agent of tularemia, a zoonotic systemic disease (McCoy and Chapin, 1912). There are various clinical forms of this disease depending on how it is transmitted. The type A strain of *F. tularensis* is one of the most lethal to humans. It is spread by aerosols, with an infectious dose of only 10 colony-forming units (CFUs) and a mortality rate of 60% in untreated cases. For this reason, the Type A strain is classified as a Tier 1 select agent of bioterrorism, meaning it can likely be used in a bioterrorist attack (Dennis et al., 2001). *F. tularensis* is considered pathogenic because of its ability to survive within phagocytic cells and escape from the phagosome into the cytoplasm (Checroun et al., 2006). Although the exact mechanism of phagosomal escape remains unknown, studies have suggested that disruption of genes located in the *Francisella* pathogenicity island (FPI), a gene cluster coding for a functional but atypical type VI secretion system (T6SS) (Spidlova and Stulik, 2017; Clemens et al., 2018), leads to the inability of bacteria to escape the phagosome (Celli and Zahrt, 2013). Several transcription factors such as MglA/SspA (Lauriano et al., 2004; Brotcke et al., 2006; Charity et al., 2007), PigR (Brotcke et al., 2006; Charity et al., 2007; Ramsey et al., 2015), and PmrA (Mohapatra et al., 2007; Bell et al., 2010; Ramsey and Dove, 2016) have been implicated in the regulation of virulence and stress response genes, and various models have been proposed to explain FPI genes expression (Charity et al., 2007; Ramsey and Dove, 2016; Cuthbert et al., 2017). Our recent research demonstrated that the *F. tularensis* HU protein is an additional key regulator that should not be overlooked (Stojkova et al., 2018; Pavlik and Spidlova, 2022).

HU proteins have been demonstrated to have a wide range of functions, such as DNA-binding transcription factors, initiation of DNA replication, cell division, SOS response, and galactose metabolism (Bonnefoy and Rouvière-Yaniv, 1992; Preobrajenskaya et al., 1994; Aki et al., 1996; Oberto et al., 2009; Ferrándiz et al., 2018). HU protein is also essential for the survival of *Streptococcus pneumoniae* (Ferrándiz et al., 2018). Furthermore, it has been reported that HU protein regulates 8% of the genes in *Escherichia coli*, particularly those involved in adaptation to the host cell and stress response (Oberto et al., 2009). This protein is also important for the initiation of the SOS response by displacing the repressor LexA (Preobrajenskaya et al., 1994). The HU protein can affect virulence gene expression in *Salmonella enterica* serovar Typhimurium (Mangan et al., 2011), *F. tularensis* (Stojkova et al., 2018), and *Porphyromonas gingivalis* (Priyadarshini et al., 2013). Additionally, its deletion leads to a reduced growth rate and decreased type III secretion system-related gene expression in *Vibrio parahaemolyticus* (Phan et al., 2015). HU protein has been shown as well to be important for cellular motility in *Salmonella* (Mangan et al., 2011), *Xanthomonas citri* (Conforte et al., 2018), and *Cytophaga hutchinsonii* (Guan et al., 2018). It also controls the transcription of genes involved in anaerobic respiration (nitrate reductase A *narH*) in both *E. coli* (Oberto et al., 2009) and *Salmonella* (Mangan et al., 2011). Furthermore, the HU protein can be a potential target for the development of therapies against tuberculosis, as it has been demonstrated to be involved in the repression of *gal* transcription and oxidative stress response (Aki et al., 1996; Balandina et al., 2001; Bhowmick et al., 2014). It is also essential for biofilm formation and pathogenesis in *X. citri* (Conforte et al., 2018) and *C. hutchinsonii* (Guan et al., 2018). Therefore, the HU protein is essential for the regulation of numerous genes and metabolic pathways within bacterial cells, and its diverse functions explain the wide range of phenotypes in HU protein-deficient strains.

Recently, the significance of HU protein in *F. tularensis* subsp. *holarctica* FSC200 intracellular growth and virulence has been uncovered (Stojkova et al., 2018). This protein, previously well studied in Gram-negative bacteria other than *F. tularensis* (Stojkova et al., 2019), usually forms heterodimers (*Enterobacteriaceae)* and is encoded by two genes: *hupA* and *hupB*. However, when found in *F. tularensis* or *Mycobacterium tuberculosis*, HU is encoded by a single *hupB* gene and forms homodimers (Bhowmick et al., 2014; Stojkova et al., 2019; Spidlova et al., 2020). HU is crucial for the replication and virulence of the FSC200 strain in mice (Stojkova et al., 2018) and it binds double-stranded DNA and protects against hydroxyl radicals. Deletion of the *hupB* gene, which encodes HU, leads to decreased expression of PigR and most FPI proteins, as well as the downregulation of *pigR* and several FPI genes. An *F. tularensis* mutant strain lacking the *hupB* gene was attenuated both *in vitro* and *in vivo*, illustrating the importance of HU for *Francisella* virulence (Stojkova et al., 2018). Our latest study showed that arginines 58 and 61 are necessary for the HU protein DNA-binding capacity in *F. tularensis*. Moreover, we identified a potential DNA-binding motif in the HU protein, suggesting that it can bind DNA in a novel sequence-dependent manner (Pavlik and Spidlova, 2022).

Gp46 is encoded by the genome of the lytic phage SPO1 of *B. subtilis*. It has no sequence similarity to other proteins with the described function. Recently, Zhang et al. described this protein as an inhibitor of the *B. subtilis* HU protein (Zhang et al., 2022). Expression of Gp46 *in trans* in *B. subtilis* led to *B. subtilis* growth reduction, cell filamentation, and blocked chromosome segregation. This phenotype resembled that of *E. coli* HU deletion mutant. Those authors also found that Gp46 interacts with *B. subtilis* HU protein at its DNA-binding site, and they speculated that Gp46 could be a cross-species HU protein inhibitor (Zhang et al., 2022).

Here, we describe the role of Gp46 on *F. tularensis* HU protein activity and the effects of plasmid-borne Gp46 expression on the vitality and pathogenicity of *F. tularensis* subsp. *holarctica* FSC200 *in vitro*. We confirmed the inhibitory effect of Gp46 on HU protein DNA binding activity leading to decreased expression of *pigR* gene coding for one of *Francisella* FPI regulator, revealed that the growth of FSC200 expressing Gp46 *in trans* was similar to that of the HU protein deletion mutant strain, and observed that the efficiency of bacterial proliferation inside mouse macrophages was significantly reduced when compared to the wild-type strain FSC200. Thus, we confirmed its impact on *in vitro* virulence of *Francisella*.

## 2 Materials and methods

### 2.1 Bacterial strains and growth conditions

The bacterial strains and plasmids used in this study are summarized in Supplementary Table S1. The bacterial strains were cultured as previously described (Stojkova et al., 2018; Pavlik and Spidlova, 2022). McLeod agar plate enriched for bovine hemoglobin (BD Diagnostics, 212392) and IsoVitalex (BD Diagnostics, 211876) or Chamberlain medium (Chamberlain, 1965) with shaking 200 rpm at 37°C were used for *Francisella* cultivation. The *E. coli* strains were cultured in Luria Bertani (LB) broth medium or on LB agar plates. When necessary, antibiotics were used at the following concentrations: kanamycin 20 μg/mL (*F. tularensis*) or 50 μg/mL (*E. coli*).

### 2.2 Generation of Gp46 expressing constructs

#### 2.2.1 Gp46 construct preparation and expression in *Escherichia coli*

The DNA sequence coding for the bacteriophage SPO1 Gp46 protein was downloaded from NCBI.^1^ As shown graphically in Supplementary Figure S1, the complete gene fused with the sequence coding for the HA tag (hemagglutinin) was sequentially synthesized by several overlap polymerase chain reactions (PCRs) using template oligonucleotides and PCR primers listed in Supplementary Table S2. The accuracy of the final PCR product was confirmed by sequencing (Institute of Microbiology, Czech Academy of Sciences). *gp46* was cloned into the pET28b vector (Novagen) using *Nco*I and *Xho*I restriction sites and propagated in *E. coli* BL21 (DE3) (New England BioLabs, Ipswich, MA, United States).

#### 2.2.2 Expression of plasmid-borne Gp46 in *Francisella tularensis*

To propagate Gp46 in *F. tularensis*, gene *gp46* was amplified using the primers gp46pKK_Fw and gp46pKK_Rev (Supplementary Table S2) using gp46pET28b as a template. The reverse primer contained sequence coding for the HA tag. Using the restriction enzymes *Nde*I and *Sac*I, the PCR product was cloned into the *Francisella* shuttle vector pKK289KmGFP (Bönquist et al., 2008) replacing the *gfp* gene. Electroporation (2,500 V, 600 Ω. 25 μF) was used to insert the final product into *F. tularensis* strain FSC200, and the strain was denoted as FSC200/Gp46. Expression of Gp46 protein was verified using Western blot and anti-HA antibody (Abcam, Ab128131) (Supplementary Figure S4).

### 2.3 Molecular docking and molecular dynamics simulation

*F. tularensis* subsp. *holarctica* FSC200 strain HU protein (FTS_0886, AFT92728, CP003862.1) structure was predicted as previously described (Pavlik and Spidlova, 2022). The predicted structure of *F. tularensis* HU, experimental crystal structure of Gp46 (Bacteriophage SPO1 protein Gp46; RCSB:7BY7) (Zhang et al., 2022), and DNA 16 bp long corresponding to the DNA-binding motif (Pavlik and Spidlova, 2022) of *F. tularensis* HU found inside the *pigR* gene were used in molecular docking studies of protein–protein or protein–DNA complexes using the HDOCK server (Yan et al., 2020) with default parameters. The best matching models were visualized using the BIOVIA Discovery Studio Visualizer (Dassault Systèmes BIOVIA, San Diego, CA, USA) and used in further simulation analyses.

Molecular dynamics simulation (MDS) was performed as previously described (Pavlik et al., 2023). MDS was achieved using GROMACS v2020.1 (van der Spoel et al., 2005; Abraham et al., 2015; Lemkul, 2019). Force fields CHARMM36 all-atom (Vanommeslaeghe et al., 2010; Grosdidier et al., 2011; Vanommeslaeghe et al., 2012; Vanommeslaeghe and MacKerell Jr, 2012; Soteras Gutiérrez et al., 2016) and AMBER (Wang et al., 2000) were used for protein or protein–DNA complex stability analyses, respectively. After constructing the topology complex, it was placed in a cubic box and solvated with water. Na^+^ and Cl^−^ ions were added to neutralize the system. Poor contact between the atoms was minimized by performing energy minimization with 50,000 steps. The system was equilibrated using standard constant volume and temperature (NVT) and constant pressure and temperature (NPT) parameters. MDS analysis was run for 50 ns, and the stabilities of the complexes were analyzed using the root-mean-square deviation (RMSD). Radius of gyration (Rg) was used to measure the compactness of the complex.

### 2.4 Purification of *Francisella tularensis* HU

*F. tularensis* subsp. *holarctica* FSC200 carrying the HU protein labeled with an HA tag (HU_HA) (Pavlik and Spidlova, 2022) was cultured overnight in Chamberlain’s medium supplemented with kanamycin (20 μg/mL). The pellet was resuspended in Tris-buffered saline (TBS) and lysed using a French press (16,000 psi, three times). The clear lysate was mixed with anti-HA agarose resin (Thermo Fisher Scientific, 26,181) and incubated at 4°C overnight with rotation. HU_HA proteins bound to the resin were washed three times with TBS supplemented with 0.05% Tween20 and eluted with 3 M NaSCN. Buffer exchange was performed using Zeba Spin Desalting Columns (Thermo Fisher Scientific, 89882). The protein was stored in buffer containing 150 mM NaCl and 50 mM Tris at pH 7.5. The concentration of the protein samples was determined using a Qubit assay (Invitrogen, Q33211).

### 2.5 Purification of Gp46

*E. coli* BL21 (DE3) carrying gp46pET28b was cultured in Luria-Bertani (LB) medium supplemented with kanamycin (50 μg/mL) at 37°C overnight. The next day, the bacterial culture was diluted 1:100 in fresh LB medium with kanamycin and cultured until reaching OD_600 nm_ = 0.4–0.8, subsequently isopropyl β-D-thiogalactoside (400 μM) was used to induce protein expression. After 4 h, the bacteria were pelleted, resuspended in 2 mL of TBS, and lysed using a French press (16,000 psi, three times). The clear lysate was applied onto an anti-HA agarose resin (Thermo Fisher Scientific, 26181) and incubated at 4°C overnight with rotation. After three washes with TBS supplemented with 0.05% Tween20, bound Gp46 protein was eluted using 3 M NaSCN. Buffer exchange was performed using Zeba Spin Desalting Columns (Thermo Fisher Scientific, 89882). The protein was stored in buffer containing 150 mM NaCl and 50 mM Tris at pH 7.5. To minimize the possibility of *E. coli* HU protein co-purification, anion exchange chromatography was performed using Macro-Prep DEAE Support (Bio-Rad, 1560020). The concentration of the protein samples was determined using a Qubit assay (Invitrogen, Q33211).

### 2.6 Electrophoretic mobility shift assay (EMSA)

The inhibitory effect of Gp46 on the interaction between the HU protein and DNA was analyzed using the EMSA method. HU protein (500 ng) was incubated with 100 ng of DNA corresponding to either the 477 bp sequence upstream of the *pigR* gene, the *pigR* gene itself, or the sequence upstream of the gene encoding IL1β with or without Gp46 (0–4,000 ng). All selected DNA sequences contain DNA-binding motif of the HU protein. Reaction mixtures were incubated in binding buffer (20 mM Tris–HCl, pH 8, 0.1 mM EDTA-Na_2_, 50 mM KCl, 10 μg/mL bovine serum albumin, 5% glycerol, and 0.1 mM DL-dithiothreitol) for 20 min at 4°C. The samples were loaded onto a 1% tris-borate-EDTA (TBE) agarose gel and separated by electrophoresis (0.33× TBE, 50 V, 240 min). The complexes were visualized using UV and SYBR®Safe DNA Gel Stain (Invitrogen, S33102).

### 2.7 Isolation of RNA and RT-PCR

RNA from FSC200, FSC200/ΔHU, and FSC200/Gp46 strains was isolated from Chamberlain medium cultures of OD_600_ 0.7 using RNeasy Mini Kit (Qiagen, 74106) according to the manufacturer’s instruction. Obtained RNA was treated with DNase I (Thermo Scientific, EN0521). Aliquots of RNA were used for reverse transcription and the obtained cDNA was used for PCR amplification of target genes (*rpoA*, *pigR*, and *hupB*) using appropriate primers (Supplementary Table S2). Samples were analyzed by agarose gel electrophoresis and intensities of bands were determined by ImageJ.

### 2.8 Standard and stress growth kinetics

*F. tularensis* strains (FSC200, FSC200/ΔHU, FSC200/pKK289, and FSC200/Gp46) were grown overnight in Chamberlain’s medium (when appropriate supplemented with kanamycin 20 μg/mL) at 37°C and 200 rpm. The next day, bacterial cultures were diluted in fresh Chamberlain’s medium (for standard growth curve) or in Chamberlain’s medium supplemented with CuCl_2_ (Sigma Aldrich, 222,011) to a final concentration of 20 μM (for oxidative stress growth curve) to OD_600_ = 0.1 and 200 μL aliquots of the suspensions were applied to a 96-well plate in pentaplicates. Pure Chamberlain’s medium was used as blank. The remaining wells were filled with water to prevent evaporation. Growth kinetics were determined by measuring optical density at 600 nm using a BioTek Synergy H1 microplate reader (Agilent). The experiment was repeated four times.

### 2.9 *In vitro* proliferation in bone marrow-derived macrophages

Mouse bone marrow cells were isolated from femurs of female Balb/c mice 6–10 weeks old (Velaz, Prague, Czech Republic) and differentiated into bone marrow-derived macrophages (BMMs), as described previously (Spidlova et al., 2018; Stojkova et al., 2018). BMMs were infected with *F. tularensis* strains (FSC200, FSC200/ΔHU, FSC200/Gp46, and FSC200/pKK289) at a multiplicity of infection (MOI) of 50. After 30 min of incubation, extracellular bacteria were killed using gentamicin (5 μg/mL). At specific time points (1, 6, 18, and 24 h) after infection, cells were lysed with 0.1% sodium deoxycholate. The lysates were plated on McLeod agar plates at appropriate dilutions. The plates were then incubated at 37°C for several days. The number of viable intracellular bacteria was determined by counting the colony-forming units (CFU). The experiment was performed in triplicate for each strain and was repeated twice.

### 2.10 Infection of the mouse model

Groups of five BALB/c mice were inoculated subcutaneously with one of the *F. tularensis* strains (FSC200, FSC200/ΔHU, or FSC200/Gp46) at an infection dose of 3 × 10^2^/mouse. The mice were monitored for signs of illness, and morbidity was recorded.

### 2.11 Statistical analysis

Statistical significance was determined using GraphPad Prism version 9 (GraphPad Software, La Jolla, CA, United States). The level of significance was defined using two-way ANOVA followed by Bonferroni’s multiple comparison test, unless otherwise indicated. *p*-values are indicated as follow: *p* < 0.05 *, *p* < 0.01 **, *p* < 0.001 ***, *p* < 0.0001 ****.

## 3 Results

### 3.1 Molecular docking and molecular dynamics simulation of Gp46 and *Francisella* HU protein

**Figure 1:**
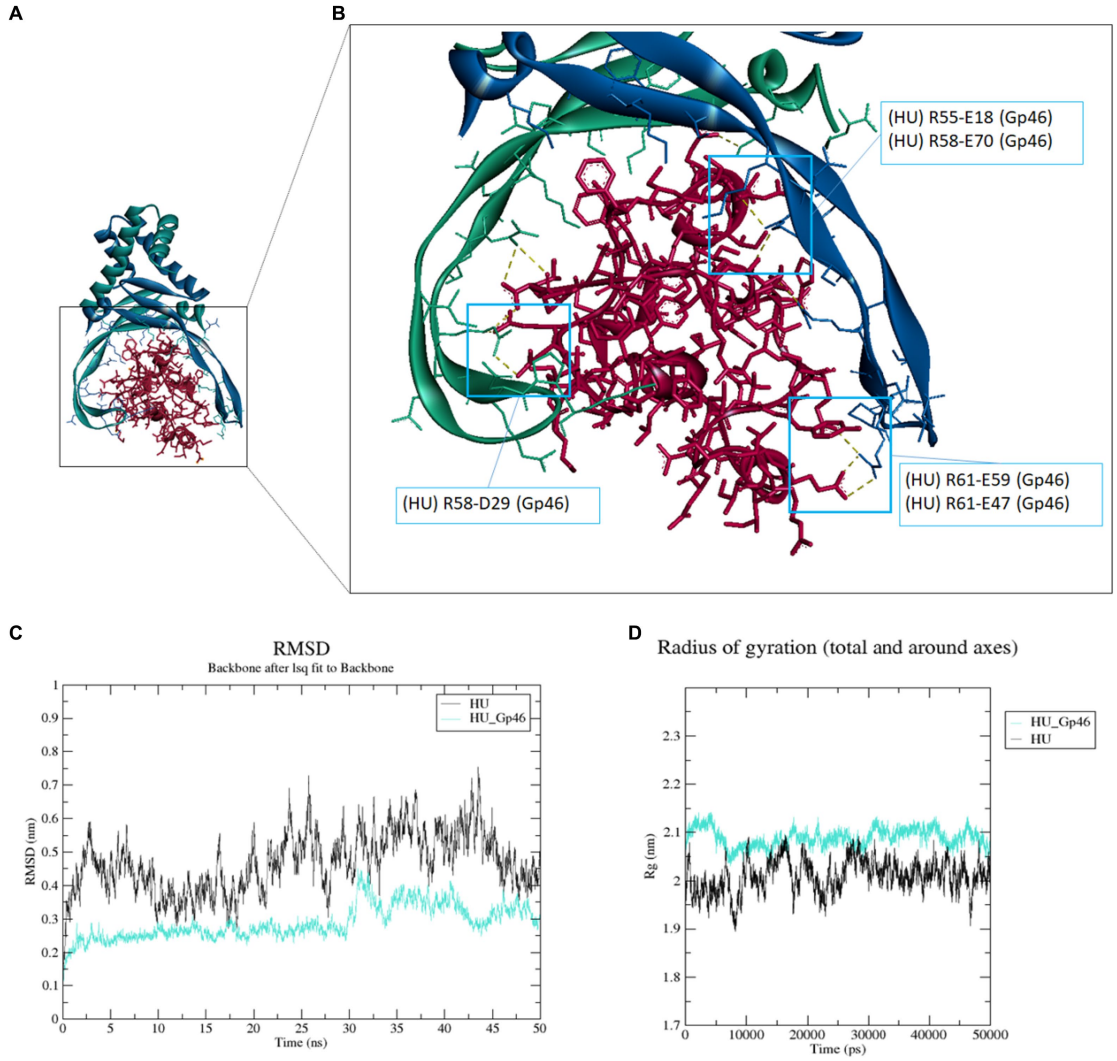
Molecular docking and molecular dynamics simulation of Gp46 and HU proteins. **(A–B)** Molecular docking of *F. tularensis* HU protein (blue-green dimer) and Gp46 (red) **(A)** and detailed view of site of interaction and prediction as to the most probable amino acid of interaction (HU protein: R55, R58, and R61) **(B)**. **(C–D)** Molecular dynamics simulation. **(C)** RMSD analysis of HU protein–Gp46 complex stability, **(D)** radius of gyration reflecting compactness of HU protein–Gp46 complex.

The structure of HU protein was predicted using the HDOCK server (Yan et al., 2020) as described previously (Pavlik et al., 2023). The bacteriophage SPO1 protein Gp46 structure was downloaded from Protein Data Bank (PDB:7BY7) (Zhang et al., 2022). We used the HDOCK server to model and predict the structure of *Francisella* HU protein and Gp46. We found that Gp46 was bound to the HU protein (Figure 1A) at the same location as in the study of *B. subtilis* HU protein and Gp46 (Zhang et al., 2022). We analyzed the most probable sites of interaction using the BIOVIA Discovery Studio Visualizer and predicted three arginine residues of the HU protein that are necessary for DNA-binding capacity (R55, R58, and R61) as amino acids interacting with Gp46 (Figure 1B). Hydrogen–hydrogen-type bonds (salt bridges) were predicted at these sites (Supplementary Table S3). We also determined the stability of the HU–Gp46 complex compared with the non-complexed HU protein using root-mean-square deviation (RMSD) analysis (Figure 1C). The free HU protein dimer proved to be less stable (0.3–0.6 nm) than the HU protein dimer in complex with Gp46 (0.2–0.4 nm) when analyzed for 50 ns, thus indicating a steady interaction of these two proteins. We also determined compactness of protein or protein complexes using radius gyration analysis (Figure 1D), where HU protein dimer is more compact (note: increased values indicate a decreased protein structure compactness, thus complex flexibility is increased but exhibits less stability) than HU-Gp46 complex, suggesting Gp46 disorganizes the stability and consistency of the HU protein.

### 3.2 Gp46 Binds *Francisella* HU protein and abolishes its DNA-binding capacity

**Figure 2:**
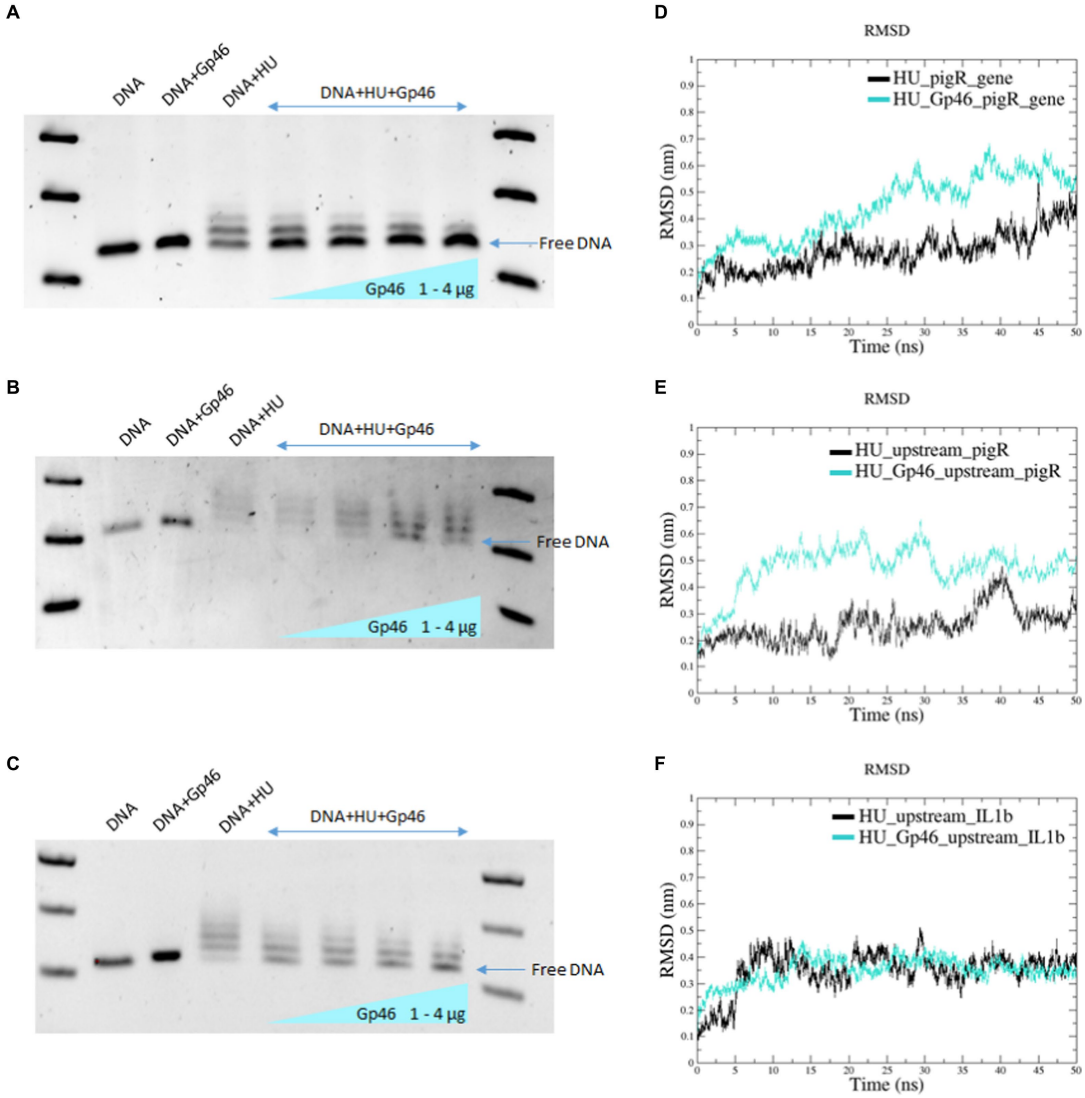
Gp46 displaces DNA from HU protein. **(A–C)** EMSA experiments using Gp46, HU, and PCR-amplified fragments of DNA [*pigR* gene **(A)**, sequence upstream of *pigR* gene **(B)**, and sequence upstream of *IL1β* gene **(C)**]. **(D–F)** RMSD analyses of complex stability using different DNA sequences (*pigR* gene **(D)**, sequence upstream of *pigR* gene **(E)**, and sequence upstream of *IL1β* gene **(F)**.

In our previous studies, we demonstrated that *F. tularensis* HU protein binds DNA and identified a *Francisella* HU protein’s specific DNA-binding motif (Pavlik and Spidlova, 2022). Using molecular docking and MDS, we showed that Gp46 and *Francisella* HU proteins formed a stable complex (Figure 1C). Next, we used the EMSA method to determine whether the Gp46 protein could displace DNA from the HU protein-binding site and occupy that site. As tested DNA we used three PCR-amplified fragments of DNA containing HU protein-binding motif/s (Pavlik and Spidlova, 2022). These PCR products corresponded to the *pigR* gene, a sequence upstream of the *pigR* gene, and a sequence upstream of the *IL1β* gene. This experiment confirmed that HU protein binds these DNA fragments (Figures 2A–C; lane DNA + HU) and that as the amount of Gp46 increased, the ability of HU protein to bind DNA decreased (Figures 2A–C; lanes DNA + HU+ Gp46, [1–4 μg of Gp46]), suggesting competition between DNA and Gp46 in HU protein DNA-binding site. Free DNA bands (blue arrows in Figure 2) densities were measured using ImageJ to show increasing amount of free DNA in the presence of Gp46 (Supplementary Figure S2). The results obtained were validated by RMSD analyses, which showed that in all cases, the complex “HU protein–DNA” was more stable than the complex “HU protein–Gp46–DNA” (Figures 2D–F), despite RMSD analysis of HU-Gp46-upstream_IL1β showed very similar values (Figure 2F) of stability, we suggest that replacing of HU protein can occur very quickly when focused on the first 5 ns. Our results corroborate that Gp46 acts as an inhibitor of *Francisella* HU DNA-binding capacity.

### 3.3 Gp46 affects transcription levels of *pigR* and *hupB*

**Figure 3:**
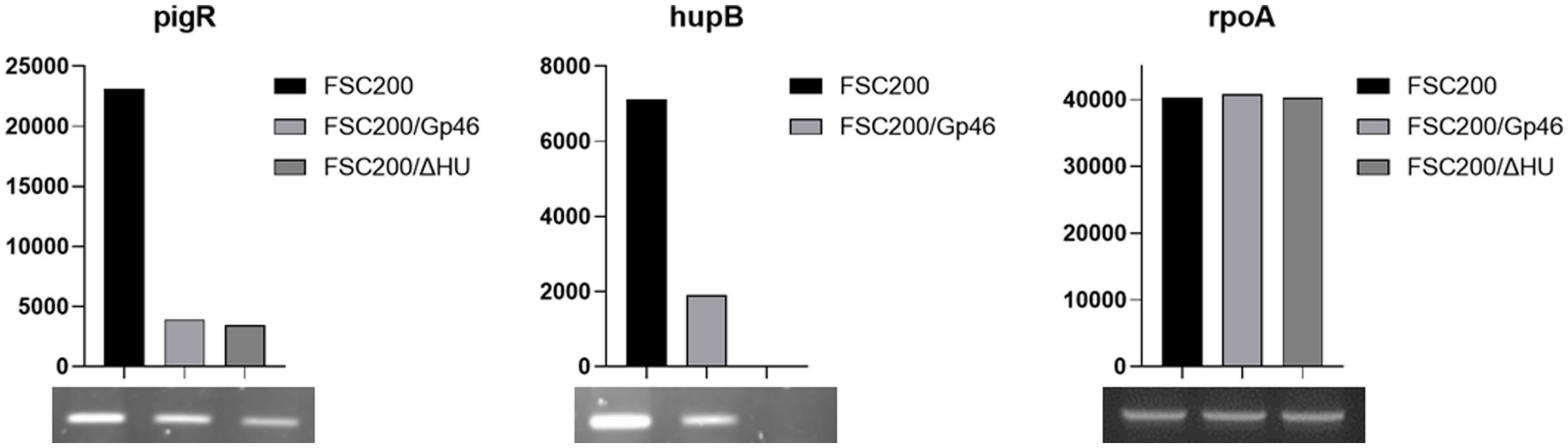
Semi-quantitative RT-PCR demonstrates decreased transcription level of *pigR* and *hupB* gene in FSC200/Gp46 and FSC200/ΔHU strains. Samples were analyzed by gel electrophoresis and intensities of bands were determined by ImageJ. Transcription level of *rpoA* as a control remained unchanged.

In EMSA experiment we have shown that Gp46 is able to block HU protein binding to dsDNA upstream *pigR* gene. In order to validate if this interaction could affect expression of *pigR* we used reverse transcription-PCR. As shown in Figure 3, plasmid-borne expression of Gp46 led to the decreased expression of *pigR* comparable to that in deletion mutant strain FSC200/ΔHU, which is in contrast to FSC200 strain. Moreover, we found out, that Gp46 alters the expression of *hupB* gene as well. The transcription level of *rpoA* which was used as a control based on previous experiments (Stojkova et al., 2018) remained unchanged.

### 3.4 Bacteriophage SPO1 protein Gp46 alters the growth of *Francisella tularensis* similarly as does the HU protein deletion strain

**Figure 4:**
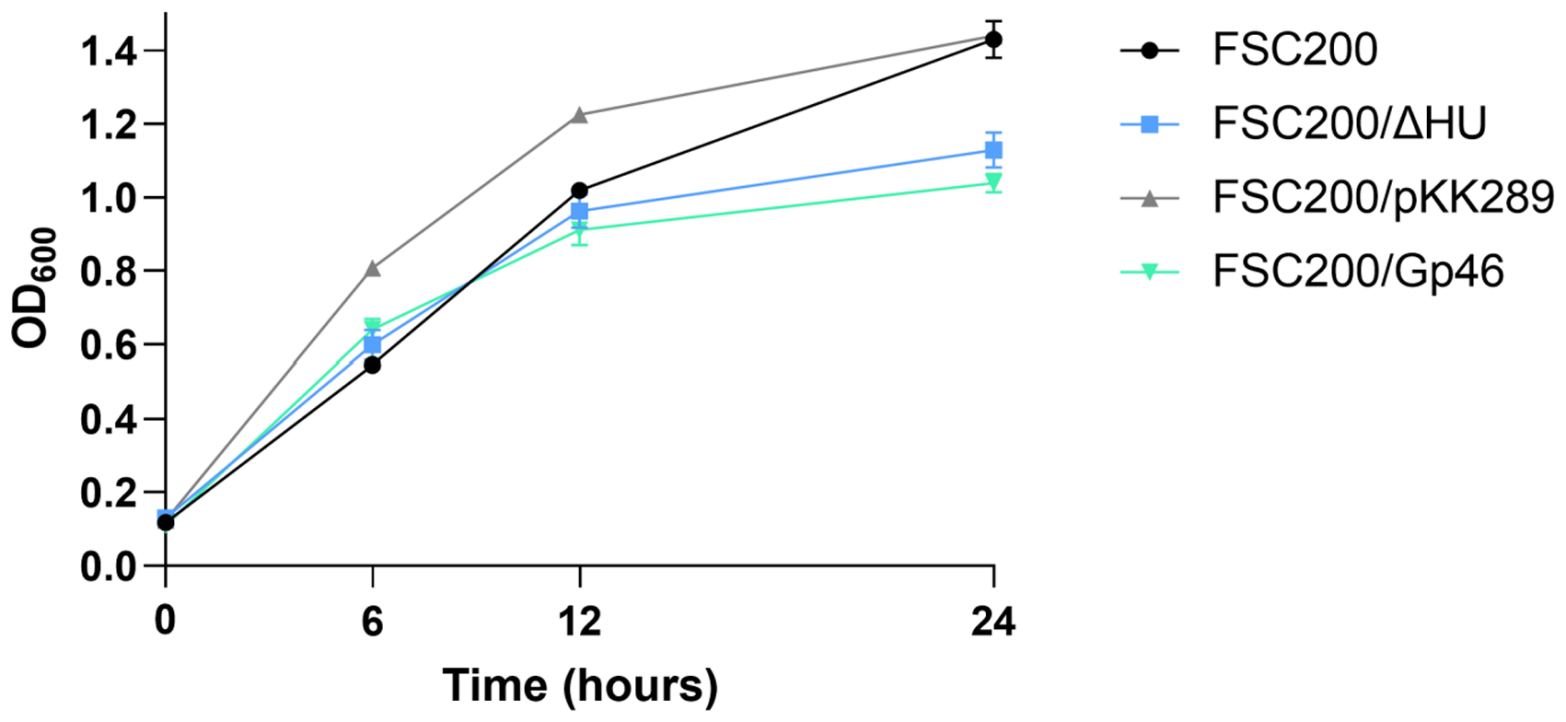
Growth kinetics of *F. tularensis* strains. Bacteria were grown in Chamberlain’s medium (when appropriate with kanamycin at a concentration of 20 μg/mL) at 37°C for 24 h in a 96-well plate. Growth kinetics were determined by measuring the optical density at 600 nm using a Synergy H1 microplate reader.

To verify the biological relevance of Gp46 as an inhibitor of *F. tularensis* HU protein, we prepared a wild-type strain *F. tularensis* FSC200 expressing plasmid-borne Gp46 (FSC200/Gp46). First, the growth kinetics of FSC200/Gp46 were compared to those of the wild-type FSC200, deletion mutant FSC200/ΔHU strains, and a strain FSC200/pKK289 expressing empty kanamycin resistant plasmid. Previously, we reported that the growth of the deletion mutant strain FSC200/ΔHU was comparable to that of the wild-type strain FSC200, even if the mutant strain entered the stationary phase of growth earlier (Stojkova et al., 2018). Here, we show that the growth of FSC200/Gp46 resembles that of the deletion mutant strain (Figure 4), which is in line with our hypothesis that Gp46 inhibits the HU protein.

### 3.5 *Francisella* wild-type strain expressing Gp46 shows similar growth defect in oxidative stress conditions as does the HU protein deletion strain

HU protein is known to protect the bacterium against oxidative stress. *Francisella* deletion mutant strain FSC200/ΔHU is unable to withstand oxidative stress conditions, as we have previously published (Stojkova et al., 2018). Therefore, we tested whether the wild-type strain expressing Gp46 shows the same phenotype under stressful growth conditions. We used CuCl_2_ to induce the production of reactive oxygen species (ROS) and therefore oxidative stress growth conditions.

**Figure 5:**
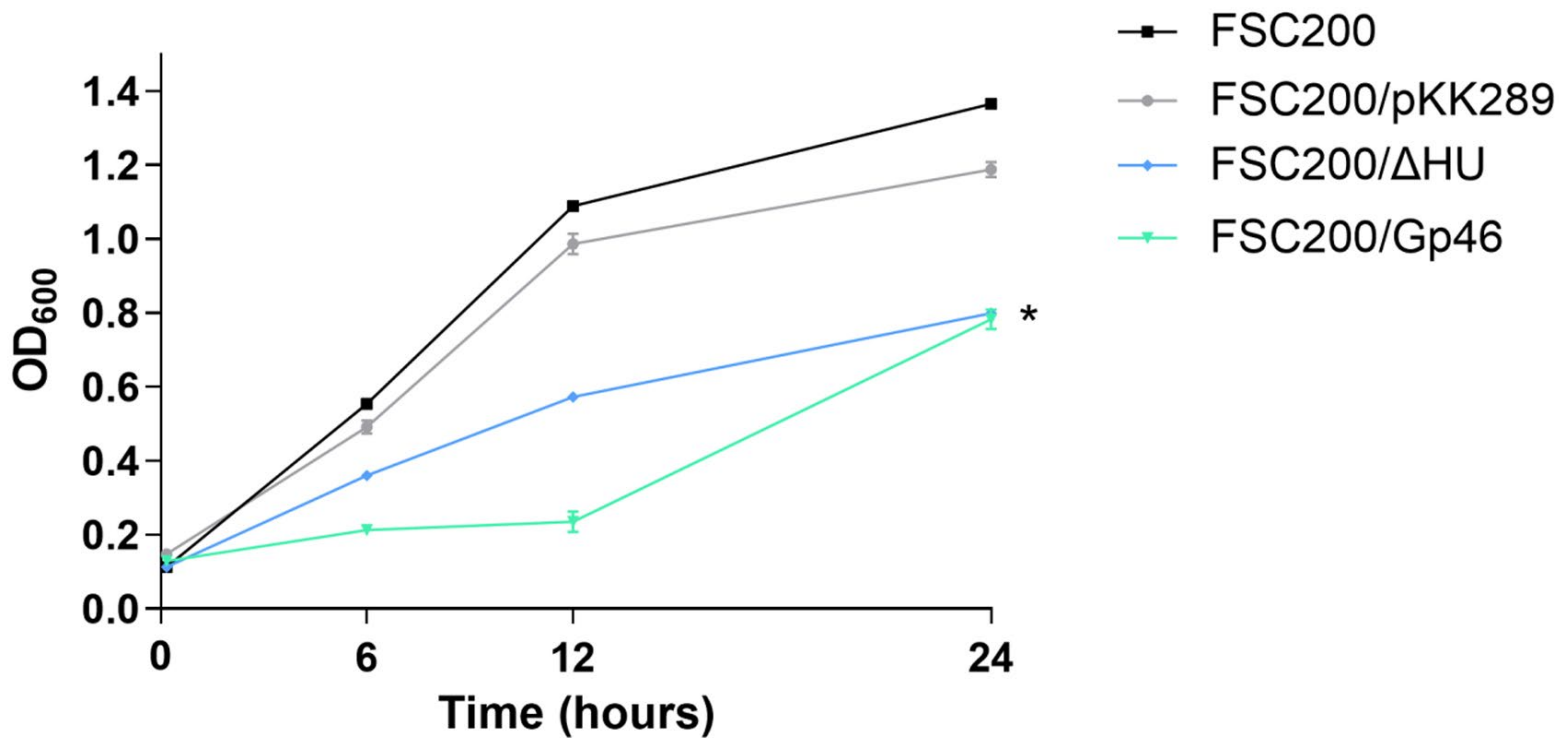
Oxidative stress growth curves. FSC200, FSC200/ΔHU, FSC200/Gp46, and FSC200/pKK289 strains were cultured in Chamberlain’s medium supplemented with CuCl_2_ at a final concentration of 20 μM for 24 h in a 96-well plate. Growth kinetics were determined by measuring the optical density at 600 nm using a Synergy H1 microplate reader.

We found that the wild-type strain expressing Gp46 fails to grow adequately under conditions of oxidative stress, similar to the FSC200/ΔHU deletion mutant strain (Figure 5). Together with the other results of this study, we demonstrate that Gp46 inhibits HU protein action in *Francisella*. Additionally, using a control, wild-type strain carrying an empty kanamycin-resistant plasmid, we show here that this growth defect is not caused by the antibiotic or the plasmid used.

### 3.6 Gp46 Inhibits intracellular replication of *Francisella* in macrophages

**Figure 6:**
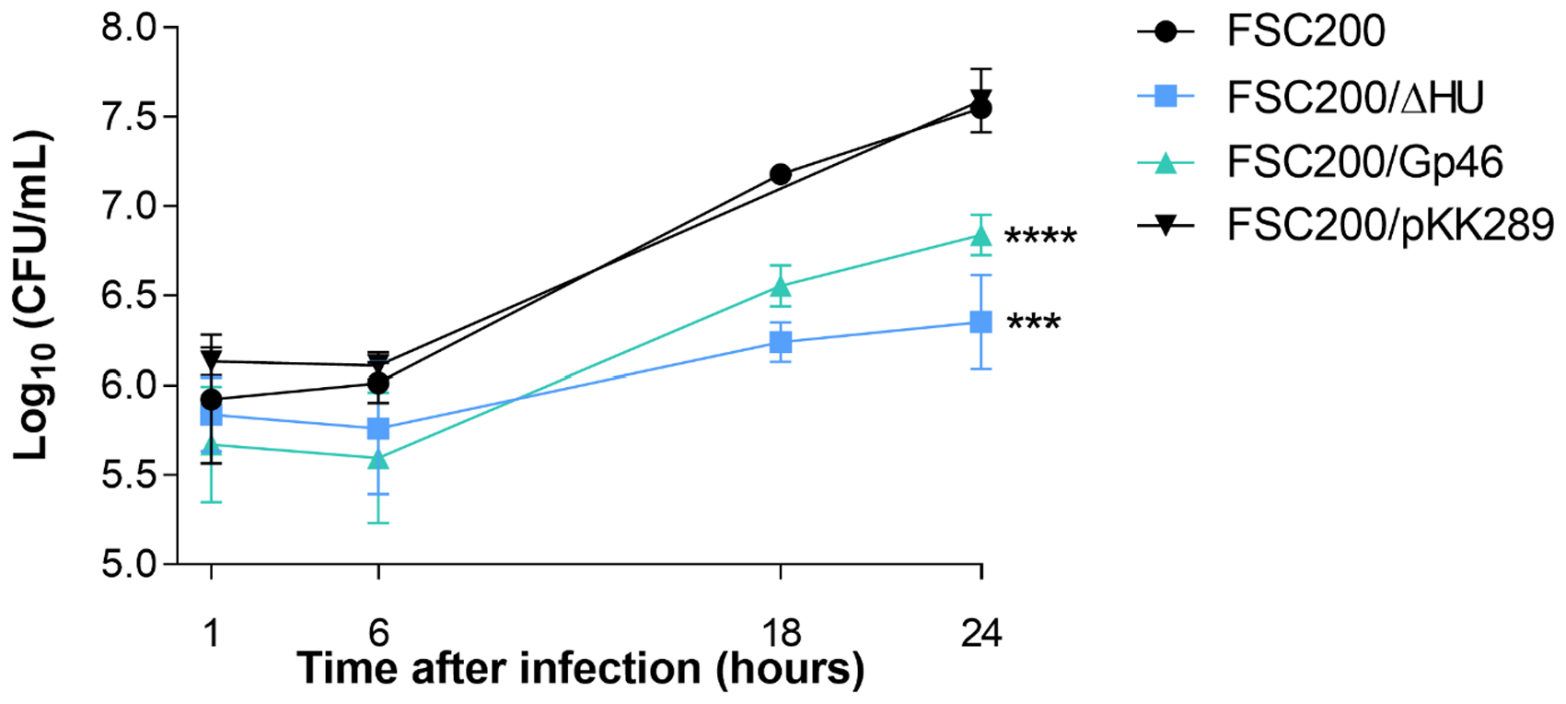
Gp46 inhibits intracellular replication of *Francisella* in macrophages. Bone marrow-derived macrophages were infected with the FSC200, FSC200/ΔHU, FSC200/Gp46, and FSC200/pKK289 strains at a multiplicity of infection of 50. *Francisella* multiplication was determined in selected time intervals of 1, 6, 18, and 24 h post-infection. Statistical analysis comparing the intracellular replication of FSC200/ΔHU and FSC200/Gp46 strains with strain FSC200 was used.

Our earlier study demonstrated that the deletion mutant strain lacking the HU protein replicated in mouse marrow-derived macrophages less effectively than the wild-type strain (Stojkova et al., 2018). If Gp46 blocks the functioning of the HU protein, we expected that the replication efficiency of the FSC200/Gp46 strain would be reduced in comparison to that of the wild-type strain and approximately the same as that of the deletion mutant strain. BMMs were infected with the FSC200, FSC200/ΔHU, FSC200/Gp46, and FSC200/pKK289 strains at an MOI of 50. Time intervals of 1, 6, 18, and 24 h post-infection were selected for counting of *Francisella* multiplication. The logarithm of the number of viable bacteria (CFU/mL), depending on the time post-infection, is shown in Figure 6. The graph clearly demonstrates that the proliferation efficiency of FSC200 expressing plasmid-borne Gp46 was affected. The strain replicated less effectively than the wild-type strain, and the similarity with the deletion mutant strain was obvious. As was mentioned above the FSC200/Gp46 multiplication defect is not linked to the general growth defect. This is the first report to indicate that Gp46 diminishes the intracellular replication of *F. tularensis* in BMMs by inhibiting DNA binding with the HU protein.

## 4 Discussion

Based on a recent study of *B. subtilis* SPO1 bacteriophage protein Gp46 as a potential universal inhibitor of bacterial HU proteins (Zhang et al., 2022), we decided to verify whether Gp46 can also inhibit *F. tularensis* HU protein and thus could be used as an effective treatment against tularemia. We analyzed the possible interaction between *F. tularensis* HU protein and Gp46 using bioinformatic approaches. Using docking simulations (Figure 1), we showed that the *Francisella* HU protein interacts with Gp46 at the same location as the *B. subtilis* HU protein. Our analysis showed that the most stable non-bond types of interaction between the HU protein and Gp46 are predicted in amino acids that are necessary for the ability of HU proteins to bind DNA (R55, R58, and R61) (Figure 1).

Subsequently, RMSD analysis confirmed the greater stability of the HU protein–Gp46 complex than that of the unbound HU protein dimer (Figures 1C,D). Consequently, using the EMSA method, we tested whether *in vitro* DNA-binding function of HU protein is affected by Gp46. Three different PCR-amplified DNA fragments containing the HU protein’s DNA binding motif, corresponding to the *pigR* gene (Figure 2A), a sequence upstream of the *pigR* gene (Figure 2B), and a sequence upstream of the *IL1β* gene (Figure 2C) were tested. The first two DNA fragments were chosen as possible sites of bacterial DNA, where the HU protein affects *F. tularensis* virulence. Previously, we described the downregulation of *pigR* expression in ΔHU deletion mutant strains (Stojkova et al., 2018) and downregulation of *pigR* expression in *Francisella* strain with a point mutation in the sequence coding for HU protein (Pavlik and Spidlova, 2022). Both these mutations resulted in strain attenuation, confirming the essential role of PigR and HU in pathogenesis of *Francisella*. The latter was chosen as a possible part of eukaryotic DNA, where the HU protein may affect the host immune response during *F. tularensis* infection. The HU binding motif/s (Pavlik and Spidlova, 2022) were present in all three chosen sequences. We confirmed that Gp46 abolishes the DNA-binding ability of the HU protein in all three tested cases, and thus, we suggest that it can act as an inhibitor of *F. tularensis* HU protein. These results were supported by RMSD analysis of complex stability, where we identified that the complex of HU protein–DNA was more stable than the complex of HU protein–Gp46-DNA (Figures 2D–F), thus suggesting that Gp46 can displace DNA from the DNA binding site of the HU protein.

Further, we analyzed the expression levels of *pigR* as well as *hupB* (Figure 3) in wild-type strain FSC200, strain expressing plasmid-borne Gp46 (FSC200/Gp46), and deletion mutant strain FSC200/ΔHU. Using reverse transcription-PCR we demonstrated that the expression of Gp46 in wild-type strain affects not only the transcription level of *pigR* which confirms our EMSA data (Figure 2B) but also the expression level of *hupB* gene. These results clearly confirmed the role of Gp46 as a HU protein inhibitor.

To contribute to the research on Gp46 as a cross-species bacterial inhibitor, we performed several *in vitro* and *in vivo* experiments. We compared the growth of *F. tularensis* wild-type strain FSC200, deletion mutant strain FSC200/ΔHU, FSC200/Gp46 expressing plasmid-borne Gp46, and FSC200/pKK289 expressing empty kanamycin resistant plasmid. We observed growth kinetics of FSC200/Gp46 similar to those of FSC200/ΔHU, suggesting that Gp46 inhibits the wild-type strain in a similar manner as deletion of the gene coding for HU protein (Figure 4). We also analyzed the resistance of wild-type strain expressing plasmid-borne Gp46 (FSC200/Gp46) to oxidative stress, because previously we showed that deletion of gene coding for HU protein significantly decreased the ability of bacterium to resist these stress growth conditions (Stojkova et al., 2018). In agreement with our hypothesis, that Gp46 blocks functioning of HU protein we observed increased of sensitivity to oxidative stress growth conditions in wild-type strain FSC200 expressing plasmid-borne Gp46 (FSC200/Gp46) (Figure 5).

The intracellular replication ability of strain FSC200/Gp46 was also compared to that of the wild-type FSC200 and FSC200/ΔHU strains as well as to that of wild-type strain carrying empty kanamycin resistant strain FSC200/pKK289 (Figure 6). We found that FSC200/Gp46 replicates inside BMMs, similar to the deletion mutant strain. FSC200/ΔHU and FSC200/Gp46 were significantly attenuated *in vitro* compared with the wild-type strain and FSC200/pKK289. Here, we show the first evidence of the ability of Gp46 to negatively affect *F. tularensis* intracellular replication, and thus could influence its virulence. Based on these results, we suggested that Gp46 may also reduce *F. tularensis* viability during infection in mice, and might be used as an agent for tularemia treatment. However, we were unable to confirm this hypothesis *in vivo*. Mice infected with FSC200/Gp46 died comparably to those infected with virulent wild-type FSC200 strain (Supplementary Figure S3). Although our pilot *in vivo* experiment was not successful and all tested mice died, we hypothesize that the effect of Gp46 on virulence of *Francisella* needs to be further studied. We assumed that the expression of Gp46 could suppress *F. tularensis* HU protein, leading to the same phenotype as the deletion mutant strain FSC200/ΔHU. However, in our experimental setup, we were not able to ensure proper replication of the plasmid-carrying *gp46* gene. The absence of attenuation of virulence of FSC200/Gp46 in mice could be due to a lack of need to express the plasmid-borne *gp46* gene because i) no selective conditions are used (replication of plasmid is useless without the necessity to ensure resistance to kanamycin), and ii) it would be inconvenient for the bacterium (clones that do not replicate plasmid are selected preferentially because they grow better and are virulent). Stable integration of *gp46* gene into the FSC200 chromosome would be more appropriate for further studies of *in vivo* experiments.

Although our study contributes to the idea that Gp46 can be a universal inhibitor of HU proteins among bacterial species, its use as an effective bacterial disease treatment, at least in the case of tularemia, remains open to further study, as well as in other bacterial species. So far the effect of Gp46 on *in vivo* inhibition of bacterial virulence has not been proven.

## Data availability statement

The datasets presented in this study can be found in online repositories. The names of the repository/repositories and accession number(s) can be found in the article/Supplementary material.

## Ethics statement

The animal study was approved by all experiments using mice were performed following guidelines of the Animal Care and Use Ethical Committee of the Faculty of Military Health Sciences, University of Defence, Czech Republic. The research protocol was approved by the ethics committee under project no. 121890/2021-1457 (3 May 2021). The study was conducted in accordance with the local legislation and institutional requirements.

## Author contributions

PS: Conceptualization, Data curation, Formal analysis, Investigation, Methodology, Project administration, Supervision, Validation, Visualization, Writing – original draft, Writing – review & editing. ES: Methodology, Writing – original draft. PP: Conceptualization, Data curation, Formal analysis, Investigation, Methodology, Project administration, Supervision, Validation, Visualization, Writing – original draft, Writing – review & editing.

## Footnotes

^1^ https://www.ncbi.nlm.nih.gov/nuccore/NC_011421.1?report=fasta&from=7212&to=7445
