# Supplementary material for: Bacteriophage SPO1 protein Gp46 suppresses functions of HU protein in Francisella tularensis
Source: Front Microbiol. 2023 Dec 8;14:1330109. doi: 10.3389/fmicb.2023.1330109 (PMC10753183; doi:10.3389/fmicb.2023.1330109)
Supplement: Supplementary file 1 [file Data_Sheet_1.pdf]

Supplementary Material

Bacteriophage SPO1 protein Gp46 suppresses virulence of Francisella tularensis in vitro through inhibition of HU protein action

Petra Spidlova*[1], Eliska Sokolova[1,2] and Pavla Pavlik*[1]

*** Correspondence:**

Dr. Petra Spidlova
petra.spidlova@unob.cz

Dr. Pavla Pavlik
pavla.s.pavlik@gmail.com

1 Supplementary Figures and Tables

1.1 Supplementary Figures

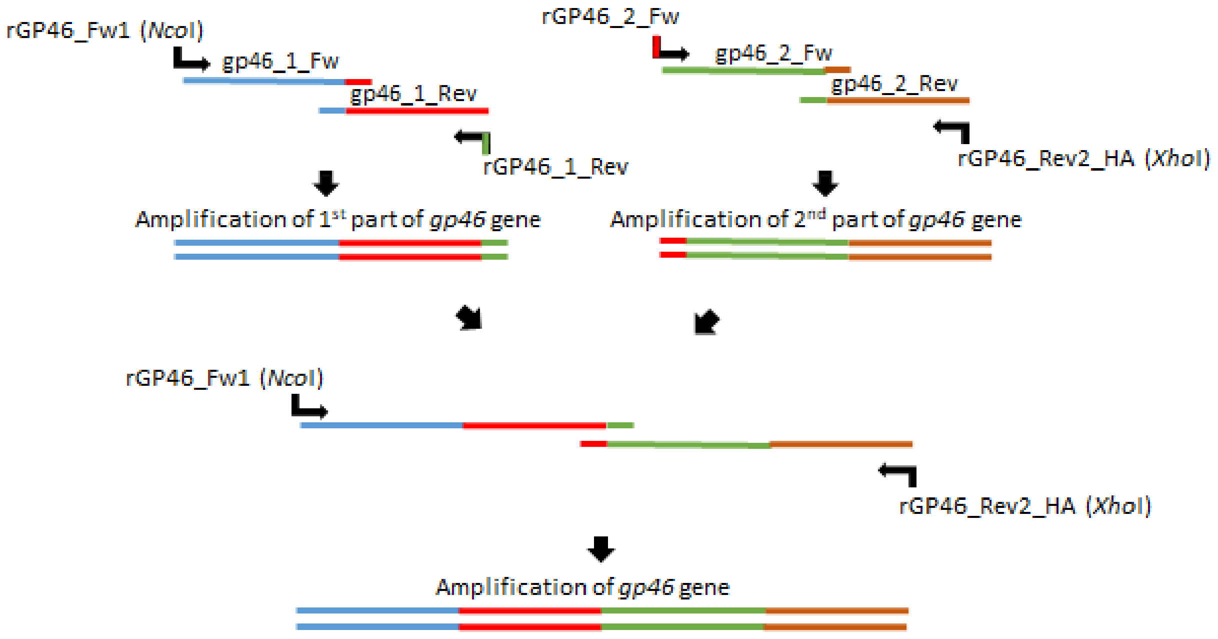

Supplementary Figure 1. Schematic representation of *gp46* gene amplification.

Oligonucleotides gp46_1_Fw and gp46_1_Rev and gp46_2_Fw and gp46_2_Rev were used as templates for overlap PCRs using the primer pairs rGP46_Fw1 (*Nco*I) – rGP46_1_Rev and rGP46_2_Fw – rGP46_Rev2_HA_(*Xho*I) to amplify the 1st and 2nd parts, of the *gp46* gene, respectively. The resulting PCR products served as templates for the final overlap PCR using the primer pair rGP46_Fw1 (*Nco*I) – rGP46_Rev2_HA_(*Xho*I) to assemble *gp46* gene.

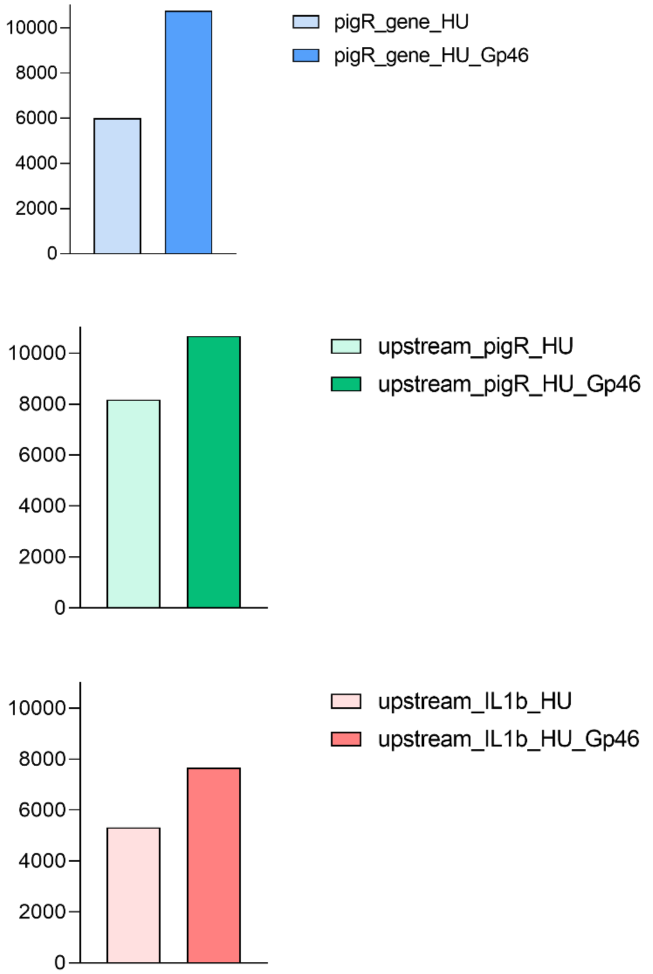

Supplementary Figure 2. Comparison of free DNA bands intensities in EMSA. Intensities of bands corresponding to free DNA in EMSA samples in the absence of GP46 (DNA+HU in Figure 2) were compared to the samples containing Gp46 at the highest concentration (DNA+HU+Gp46 in Figure2). The presence of Gp46 led to the higher amounts of free DNA in all tested DNA fragments, showing Gp46 abolishes DNA-binding capacity of *Francisella* HU protein.

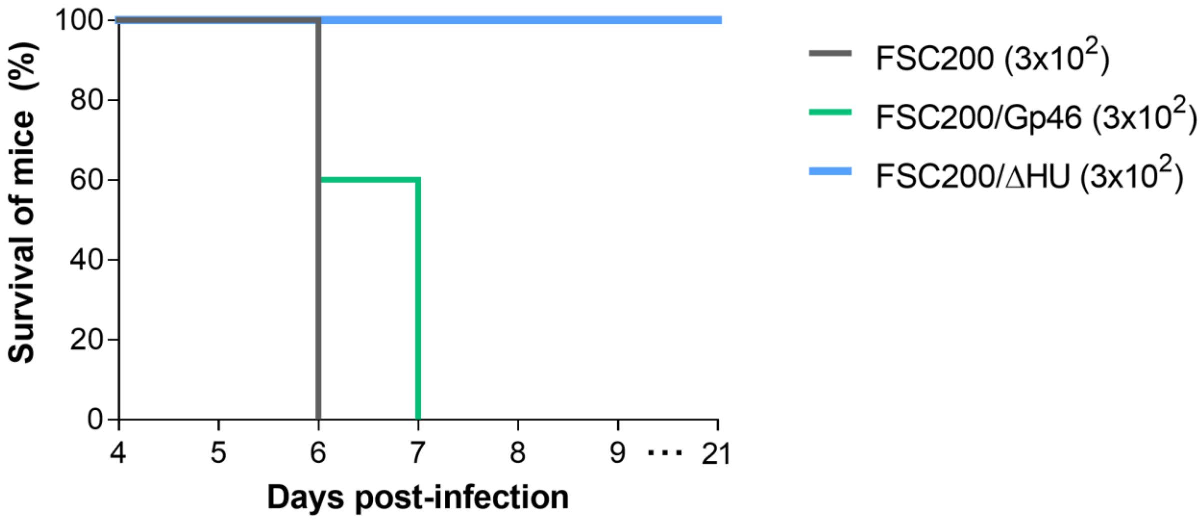

Supplementary Figure 3. Survival of BALB/c mice depending upon *Francisella* strain used. BALB/c mice were infected subcutaneously with an infection dose of 3x10^2 CFU/mouse of one of the three *Francisella* strains. Mice infected with FSC200 died six days after infection, whereas all mice infected with FSC200/ΔHU survived.

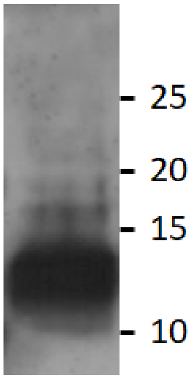

Supplementary Figure 4. Verification of Gp46 expression level in FSC200/Gp46 strain. Lysate obtained from the FSC200/Gp46 strain was used for Gp46 detection using Western blot and anti-HA antibody.

1.2 Supplementary Tables

Supplementary Table 1. Bacterial strains and plasmids used in this study.

**Bacterial strain**	**Description**	**ATB**	**Source**
E. coli BL21(DE3)/Gp46pET28b		Km	New England Biolabs
Francisella tularensis subsp. *holarctica* FSC200	Wild-type strain	-	*Francisella* Strain Collection (FSC) of the Swedish Defense Research Agency, Umea, Sweden
Francisella tularensis subsp. *holarctica* FSC200/ΔHU	Deletion mutant strain lacking the *hupB* gene (coding for HU protein)	-	[14]
Francisella tularensis subsp. *holarctica* FSC200/Gp46	Wild-type strain expressing plasmid-born Gp46	Km	This study
**Plasmid**	**Description**	**ATB**	**Application**
pKK289KmGFP	*E. coli/ F. tularensis* shuttle vector, *Ft* ori, p15a ori, groES promoter [31]	Km	Expression of Gp46 in *Francisella*
pET28b	T7 expression vector, Novagen	Km	Expression of Gp46 in *E. coli* BL21 (DE3)

Supplementary Table 2: List of primers used in this study

Primer	Sequence (5´- 3´)	Application
gp46_1_Fw	ATGATGACAGAAGACCAAAAATTTAAGTACC TGACTAAAATTGAGGAATTGGAAGCGGGATG CTTTAGTGA	Template for the first half of *gp46* gene
gp46_1_Rev	TTCTTAAGGTATTTGAGGTCACCTGTAATATC CTCCTTCGTCCAATCACTAAAGCATCCCGCTTC	Template for the first half of *gp46* gene
rGP46_Fw1_*Nco*I	ATACCATGGCCATGATGACAGAAGACCAAAA ATT	Forward primer for amplification of either *gp46* gene or first half of *gp46* gene
rGP46_1_Rev	TATGATGCCCTTCTTAAGGTATTTGAGGTCAC	Reverse primer for amplification of the first half of *gp46*
gp46_2_Fw	GGGCATCATAGAGGAGTCTATTGAATTAATCC GTGCTGTAAATGGTCTAACGTATAGTGAAGA GTT	Template for the second half of *gp46* gene
gp46_2_Rev	TAGTGGGCTGATGTCTAGTTCTTCTATAATTTC CTGAGTGAAGTCATGCAACTCTTCACTATACG TTAGACC	Template for the second half of *gp46* gene
rGP46_2_Fw	ATACCTTAAGAAGGGCATCATAGAGGAGTCT AT	Forward primer for amplification of the second half of *gp46* gene
rGP46_Rev2_HA_*Xho*I	ATATCTCGAGTTAAGCGTAATCTGGAACATCG TATGGGTATAGTGGGCTGATGTCTAGTTC	Reverse primer for amplification of either *gp46* gene or second half of *gp46* gene
gp46pKK_Fw_*Nde*I	AAACATATGATGACAGAAGACCAAAAATT	Amplification of *gp46* gene for cloning into the pKK289Km shuttle vector
gp46pKK_Rev_*Sac*I	AAAGAGCTCTTAAGCGTAATCTGGAACATCGT ATGGGTATAGTGGGCTGATGTCTAGTTC	Amplification of *gp46* gene for cloning into the pKK289Km shuttle vector
Up_IL1-b_Fw	AGTACCAGGCTCTTTTACTG	Amplification of the sequence upstream of the *il-1beta* gene for EMSA
Up_IL1-b_Rev	TCTCTGGATAAATTAAGGGTC	Amplification of the sequence upstream of the *il-1beta* gene for EMSA
PigR_Fw_*Nde*I	ATTAATATGGCGAATCAATATTCTGGAA	Amplification of the *pigR* gene for EMSA
PigR_Rev_*Sac*I	GAGCTCTCAAGATTTAGCTTTGATTACAG	Amplification of the *pigR* gene for EMSA
Up_PigR_Fw	TTCAATGTGTTTTGTTAATTTTCTC	Amplification of 477bp sequence upstream of the *pigR* gene for EMSA
Up_PigR_Rev	TATAACGAGTCTCCTTTAATTA	Amplification of 477bp sequence upstream of the *pigR* gene for EMSA
pigR_F	ATGGCGAATCAATATTCTGGAA	RT-PCR
pigR_R	CAGTCAAGATTTAGCTTTGATTA	RT-PCR
rpoA_F	GTGAGTAATAATAATTCAAAACTG	RT-PCR
rpoA_R	TTATTTTCCTTCAACTAGCTCTC	RT-PCR
hupB_F	GGATCCGAACAAGAGTGAATTAGTAAGT	RT-PCR
hupB_R	GGTACCGCTTTTACAGCGTCTTTAAGACC	RT-PCR

Supplementary Table 3.: HU_Gp46 bonds

https://docs.google.com/spreadsheets/d/1dTAsM1nX43_wHQxNe5dbK5KlmWgLA8gP/edit?usp=drive_link&ouid=105930610242123747309&rtpof=true&sd=true
